# Lichen Planus Initially Presenting as Poikiloderma: A Challenging Case Report

**DOI:** 10.7759/cureus.81641

**Published:** 2025-04-03

**Authors:** Mehad Almoqati, Renad Althobaiti, Dai Zafer, Asma S Alabbadi, Razan Alluhaibi, Khalid Al Hawsawi

**Affiliations:** 1 General Medicine, King Abdulaziz Hospital, Makkah, SAU; 2 College of Medicine, Umm Al-Qura University, Makkah, SAU; 3 Dermatology, King Abdulaziz Hospital, Makkah, SAU

**Keywords:** lichen planus, mycosis fungoides, poikiloderma, telangiectasia, violaceous papule

## Abstract

Lichen planus (LP) is a chronic inflammatory disorder that affects the skin, mucous membranes, nails, and hair. Cutaneous LP (CLP) is characterized by violaceous, polygonal, flat-topped papules and plaques that are intensely pruritic. Although it can develop on any part of the body, it most commonly affects the flexor surfaces of the wrists, lower back, and ankles. This report presents an atypical case of LP in a 33-year-old woman who initially exhibited poikilodermatous changes with bluish-gray patches, persisting for a decade. Due to the overlapping clinical and histopathological features, an extensive diagnostic workup including pan-computed tomography, lymph node biopsy, and immunohistochemistry was performed to exclude poikilodermatous mycosis fungoides. One year later, skin examination and histopathological evaluation revealed the classical features of LP, leading to a definitive diagnosis. This case highlights an unusual presentation in which poikiloderma preceded the classic clinical picture of LP. Our findings contribute to the existing knowledge of LP by emphasizing the importance of recognizing atypical presentations for accurate diagnosis and management.

## Introduction

Lichen planus (LP) is a chronic papulosquamous inflammatory disorder that is categorized into three primary subtypes, cutaneous LP (CLP), mucosal LP (MLP), and lichen planopilaris (LPP), which specifically target the scalp [1]. The prevalence of cutaneous LP ranges from 0.2% to 1% in the adult population, with most cases occurring in individuals between 30 and 60 years of age [2]. Despite the lack of a clear gender predominance, LP appears to affect adult women more frequently than men [3]. The exact etiology of LP remains undetermined, although autoimmune mechanisms are widely believed to play a role [4]. Various environmental factors, such as hepatitis C infection, sun exposure, stress, and certain medications, are also known to trigger or exacerbate the condition [5]. The hallmark features of CLP include violet-colored, polygonal, flat-topped papules and plaques that are slightly scaly and severely pruritic [6]. Common sites of involvement include the wrists, lower back, and ankles [7]. In this report, we describe the case of a 33-year-old woman with an unusual and challenging presentation of LP that initially appeared as poikiloderma. Poikiloderma refers to skin changes that include areas of thinning (atrophy), visible small blood vessels (telangiectasia), and a combination of increased and decreased pigmentation [8]. These features are nonspecific and can be seen in a range of conditions, including cutaneous T-cell lymphoma, dermatomyositis (DM), and connective tissue diseases [9]. Thus, poikiloderma can complicate the diagnosis of LP when it precedes or masks its classic clinical and histopathological features.

## Case presentation

A 33-year-old woman with a known history of allergic rhinitis presented to our clinic with a chronic skin rash that had persisted for over 10 years. The rash initially appeared on her arms and legs and then progressively extended to encompass the thighs, lower abdomen, flanks, neck, and chest area. It was moderately pruritic and mildly exacerbated by sunlight exposure.

The patient denied any associated symptoms such as arthralgia, myalgia, weakness, weight loss, fever, chills, or night sweats. She also denied a history of medication use. However, she reported significant depressive symptoms secondary to her dermatological condition.

**Figure 1:**
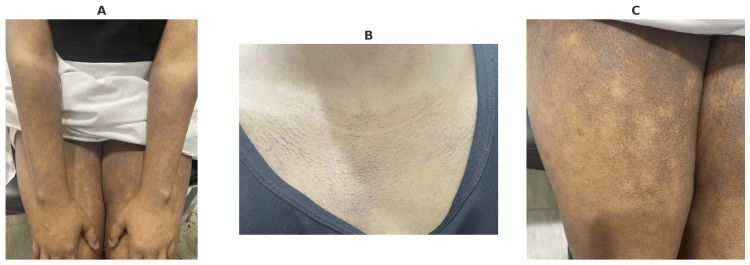
Non-blanchable bluish to grayish and erythematous patches and poikiloderma over (A) the upper and lower extremities, (B) the upper chest, and (C) the anterior thighs

During the initial visit to our clinic, a skin examination revealed non-blanchable bluish to grayish and erythematous patches over the upper and lower extremities, lower abdomen, and neck and around the breast area (poikiloderma) (Figure 1). No lesions were observed on the mucous membranes or scalp, and there was no lymphadenopathy or hepatosplenomegaly.

**Figure 2:**
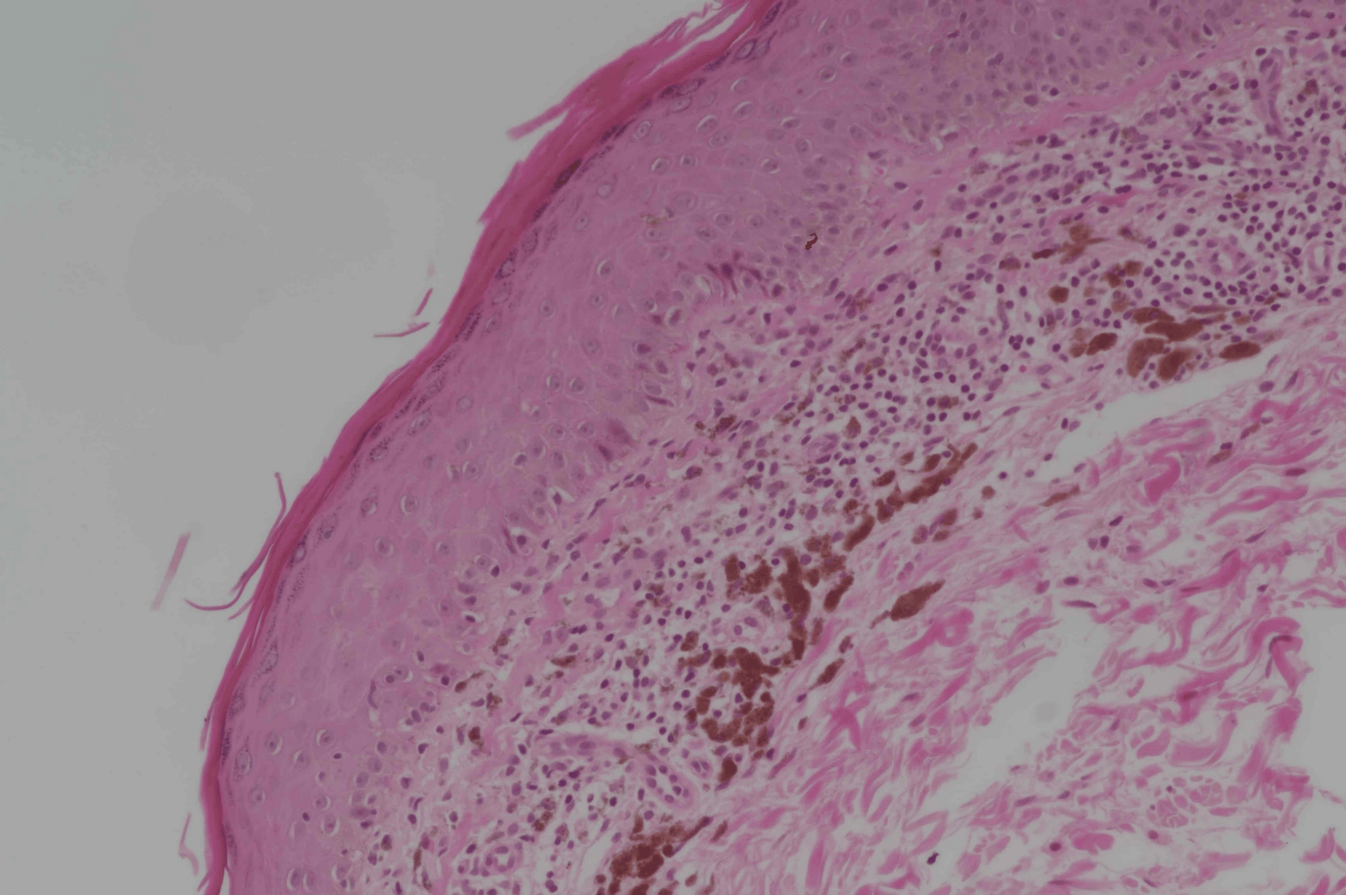
Few scattered atypical lymphoid cells in the upper dermis that infiltrated the lower epidermal layer in addition to finding melanin incontinence

**Figure 3:**
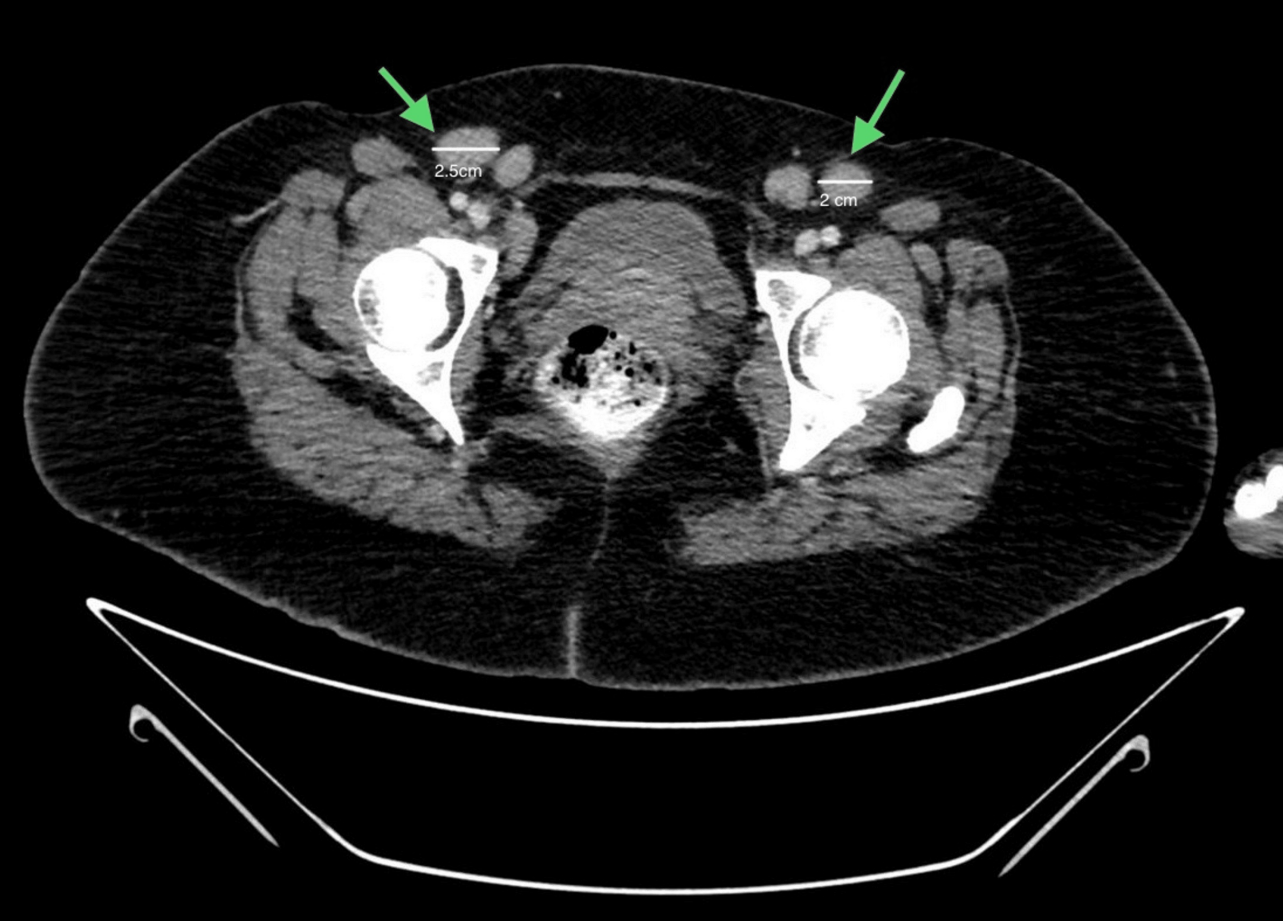
Pan-CT revealing multiple bilateral inguinal enhancing lymph nodes (scale bar: 2.5 cm) CT: computed tomography

Laboratory results, including a complete blood count and serology for hepatitis B, hepatitis C, and human immunodeficiency virus (HIV), were unremarkable. Antinuclear antibodies (ANA), lactate dehydrogenase (LDH) levels, and creatine kinase (CK) were within the normal range (Table 1). A peripheral blood smear was performed, and no atypical cells were observed. A skin biopsy was taken, revealing a few scattered atypical lymphoid cells in the upper dermis that infiltrated the lower epidermal layer, along with melanin incontinence (Figure 2). Immunohistochemical markers were sent for analysis, and the results indicated immunoreactivity to CD3, CD5, CD7, CD10, and CD20. The overall immunohistochemical profile was consistent with dermatopathic lymphadenitis, a reactive lymphoid process frequently associated with many chronic cutaneous conditions, including exfoliative or inflammatory dermatoses such as mycosis fungoides (MF). These clinicopathological findings suggested a possible diagnosis of poikilodermatous MF, which affected 70% of her body surface area. Therefore, a pan-computed tomography (CT) scan was performed, revealing multiple bilateral inguinal enhancing lymph nodes, the largest measuring up to 2.5 cm (Figure 3).

**Table 1:** Laboratory investigation CBC: complete blood count; WBC: white blood cell; Hb: hemoglobin; HIV: human immunodeficiency virus; CK: creatine kinase; LDH: lactate dehydrogenase; ANA: antinuclear antibody

Test	Patient's value	Reference range	Interpretation
CBC			
WBC (×10^9^/L)	4.22	4-11	Normal
Hb (g/L)	134	115-160	Normal
Platelet count (×10^9^/L)	431	150-450	High-normal (slightly elevated)
Infectious markers			
Hepatitis B	Negative	Negative	Normal
Hepatitis C	Negative	Negative	Normal
HIV	Negative	Negative	Normal
Muscle enzymes			
CK (IU/L)	149	26-192	Normal
Liver function			
LDH (U/L)	185	155-357	Normal
Autoimmune marker			
ANA	Normal	Negative or <1:80	Normal

**Figure 4:**
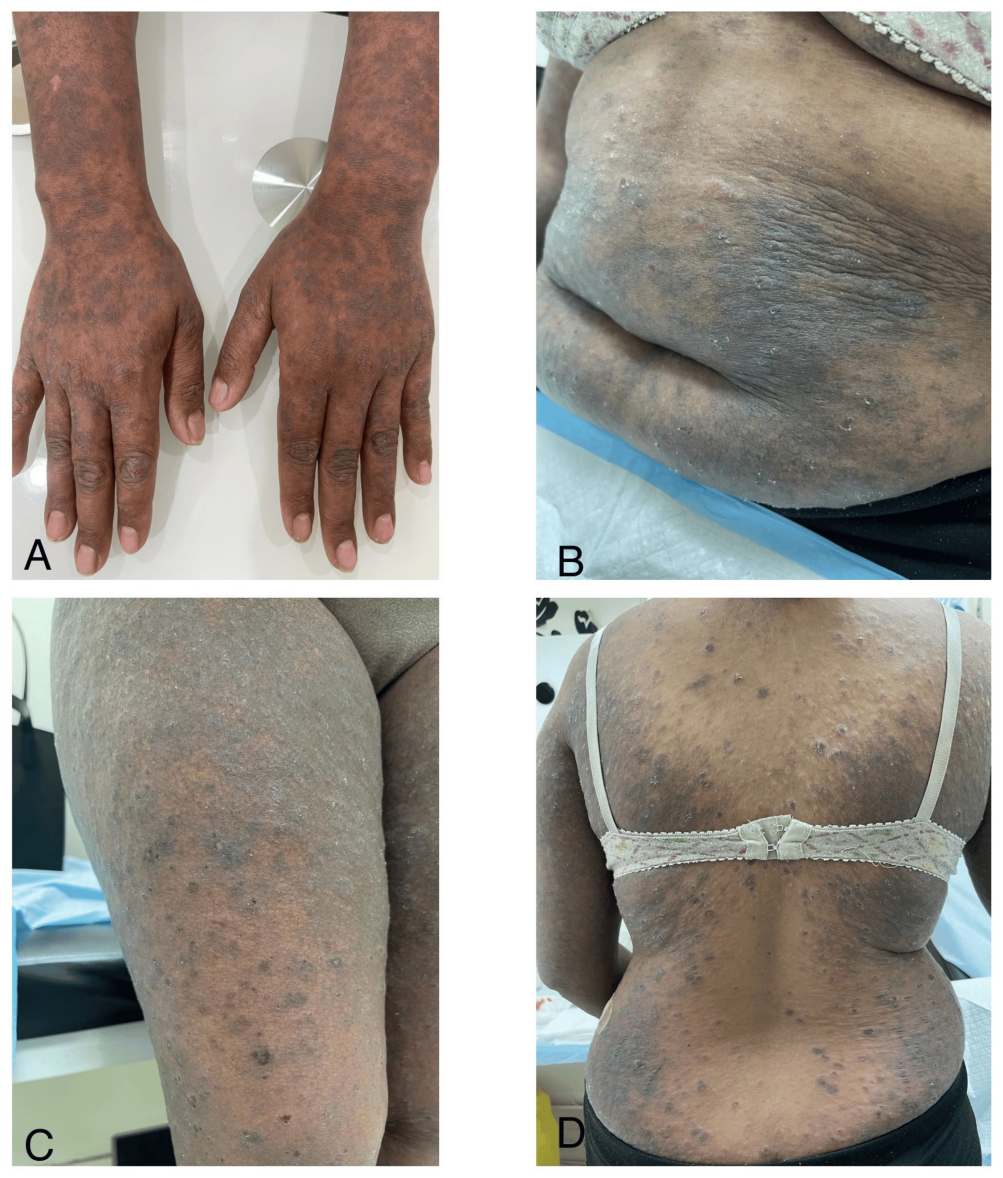
Violaceous scaly papules and plaques with excoriations along her (A) upper extremities, (B) abdomen, (C) lower extremities, and (D) back

**Figure 5:**
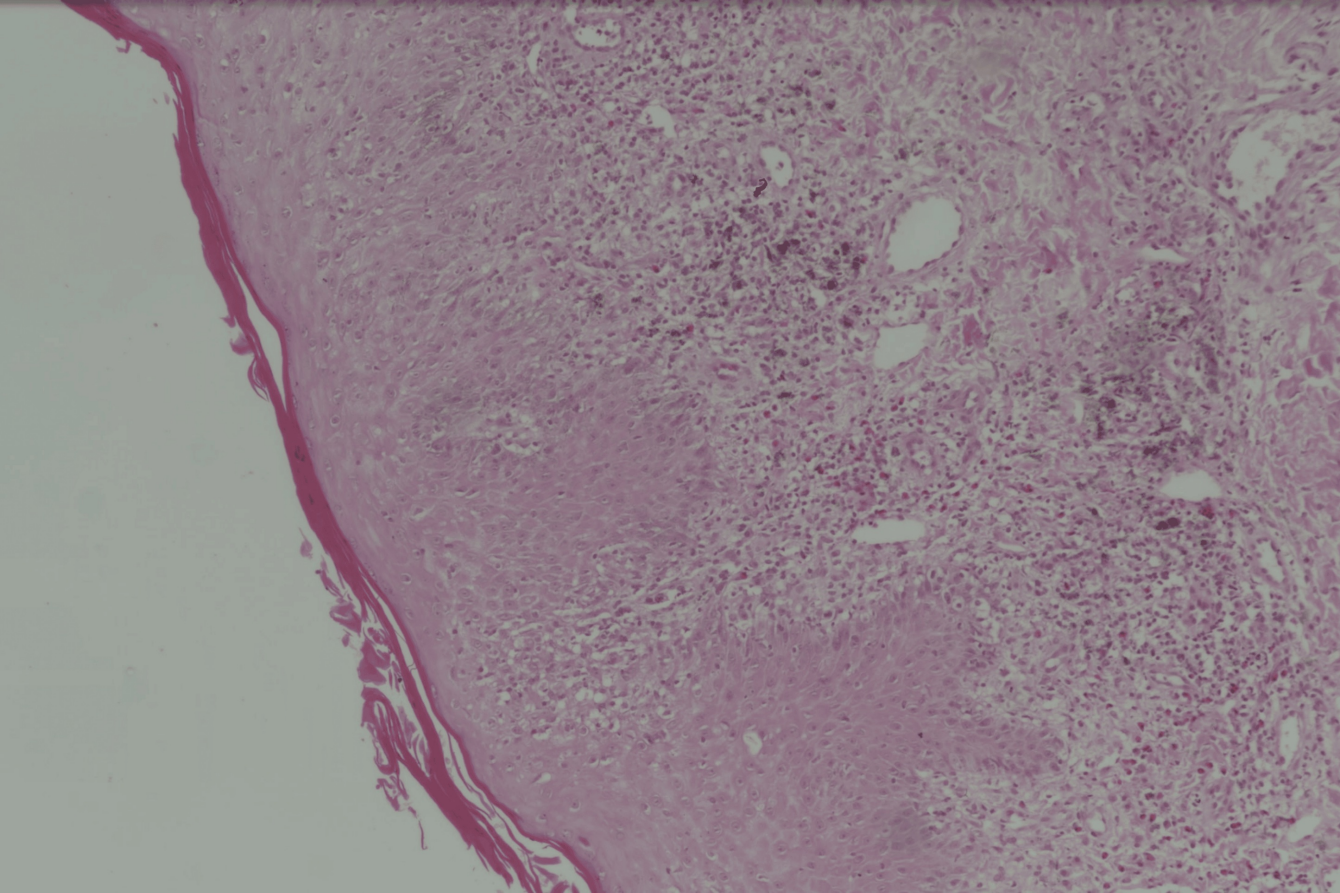
Hyperkeratosis, hypergranulosis, basal layer vacuolization, dense, band-like lymphocytic infiltrate, and melanin incontinence

A biopsy was obtained from the left inguinal lymph node, which was indicative of reactive lymphoid tissue consistent with dermatopathic lymphadenitis. The patient was prescribed topical clobetasol and underwent four sessions of narrowband ultraviolet B (NBUVB) therapy, in addition to receiving oral cetirizine (10 mg as needed), escitalopram (10 mg daily), and emollients. However, no improvement was observed due to poor compliance and missed follow-ups. One year later, the patient presented with violaceous scaly papules and plaques with excoriations along her upper and lower extremities, upper chest, and back (Figure 4). A repeat skin biopsy revealed the classic histopathological features of LP, including hyperkeratosis, hypergranulosis, basal layer vacuolization, a dense band-like lymphocytic infiltrate, and melanin incontinence (Figure 5). Repeat immunohistochemical marker analysis indicated immunoreactivity to CD3, CD5, and CD7, but the cells were non-reactive to CD20. This immunophenotype was consistent with a polyclonal T-cell infiltrate, typical of benign inflammatory dermatoses such as LP. Lack of CD20 confirms the absence of B-cell predominance. While clonality testing via T-cell receptor gene rearrangement would have added further diagnostic value, it was deferred due to the shift in clinical and histopathological findings. Thus, based on the later clinicopathological findings, a diagnosis of LP was made. Significant clinical improvement was observed, including the resolution of erythema and pruritus following the initiation of oral prednisone (30 mg daily for two weeks) combined with NBUVB therapy three times per week and topical betamethasone. The patient was subsequently scheduled for periodic follow-up appointments. She was informed of the purpose of the publication. All measures have been taken to ensure anonymity. The patient was given the opportunity to ask questions and had their concerns addressed before providing consent.

## Discussion

LP is an idiopathic, papulosquamous, subacute, or chronic inflammatory disease that affects the skin, mucous membranes, and nails [10]. In CLP, different clinical subtypes are classified based on the configuration or morphology of the lesions. These subtypes include papular (classic), hypertrophic, vesiculobullous, actinic, annular, atrophic, linear, follicular, LP pigmentosus, and LP pigmentosus-inversus [5]. While LP typically exhibits the classic clinical features, atypical presentations particularly in certain variants defined by their distinct clinical characteristics can sometimes occur, posing diagnostic and therapeutic challenges for clinicians.

Contrary to the description by Sayal et al., who reported a case of LP gradually progressing to poikiloderma over several years [11], our case demonstrated poikiloderma preceding the development of typical LP. This reverse progression suggests a potential early atypical form of LP or a related dermatological condition that subsequently evolved into classic LP. Such cases highlight the importance of observing the natural course of the disease and maintaining a broad differential diagnosis. In scenarios like these, performing a biopsy and conducting a detailed histopathological examination are crucial for confirming the diagnosis and ruling out other differential diagnoses such as poikilodermatous MF, poikiloderma of DM, drug-induced LP, or LP pigmentosus.

The possibility of poikilodermatous MF was excluded based on immunohistochemical findings, which did not demonstrate the characteristic aberrant T-cell phenotype typically seen in MF (CD4⁺, CD7⁻, CD8⁻) [12]. Additionally, poikilodermatous DM was considered in the differential diagnosis. Accordingly, CK levels were assessed and found to be within normal limits. However, the patient did not exhibit hallmark features of DM, such as a heliotrope rash or Gottron's papules. ANA result and the absence of systemic symptoms further made DM unlikely [13]. Furthermore, there was no history of ionizing radiation exposure or medication use that could have triggered drug-induced LP [14].

CLP can be a self-limiting disease, and the primary goal of treatment is to control symptoms and reduce the duration of lesions [1]. First-line treatments include topical glucocorticoids, which can be used alone or in combination with phototherapy modalities such as broadband or NBUVB phototherapy or psoralen plus ultraviolet A (PUVA) therapy [15,16]. When these options prove insufficient, systemic glucocorticoids or retinoids, such as acitretin or isotretinoin, may be considered [1]. Ensuring an individualized approach based on disease severity and patient response is key in managing such conditions.

Our patient was started on oral steroids and NBUVB phototherapy, which resulted in excellent clinical improvement. The sequence of progression from poikiloderma to classic LP in this patient underscores the need for vigilant observation and follow-up in atypical cases. It highlights the evolving nature of LP presentations and the importance of timely diagnosis and treatment in achieving favorable outcomes.

## Conclusions

Poikiloderma is a rare presenting feature of LP that requires more attention, which can be achieved by integrating medical history, physical examination, and histopathological findings. This case contributes to the understanding of atypical LP presentations by documenting a case in which poikiloderma preceded the classical picture of LP. Therefore, our report emphasizes the importance of recognizing this uncommon presentation to prevent misdiagnosis and provide proper treatment. 

## References

[1] Le Cleach L, Chosidow O (2012). Lichen planus. N Engl J Med.

[2] Boyd AS, Neldner KH (1991). Lichen planus. J Am Acad Dermatol.

[3] Weston G, Payette M (2015). Update on lichen planus and its clinical variants. Int J Womens Dermatol.

[4] Solimani F, Forchhammer S, Schloegl A, Ghoreschi K, Meier K (2021). Lichen planus - a clinical guide. J Dtsch Dermatol Ges.

[5] Gorouhi F, Davari P, Fazel N (2014). Cutaneous and mucosal lichen planus: a comprehensive review of clinical subtypes, risk factors, diagnosis, and prognosis. ScientificWorldJournal.

[6] Yogianti F, Pradipta NK (2021). Case of lichen planus with unusual features. BMJ Case Rep.

[7] Tziotzios C, Lee JY, Brier T (2018). Lichen planus and lichenoid dermatoses: clinical overview and molecular basis. J Am Acad Dermatol.

[8] Lipsker D (2003). What is poikiloderma?. Dermatology.

[9] Bloom B, Marchbein S, Fischer M, Kamino H, Patel R, Latkowski JA (2012). Poikilodermatous mycosis fungoides. Dermatol Online J.

[10] Parihar A, Sharma S, Bhattacharya SN, Singh UR (2015). A clinicopathological study of cutaneous lichen planus. J Dermatol Dermatol Surg.

[11] Sayal SK, Gupta CM, Malik AK (2001). Lichen planus induced poikiloderma. Indian J Dermatol.

[12] Olsen E, Vonderheid E, Pimpinelli N (2007). Revisions to the staging and classification of mycosis fungoides and Sezary syndrome: a proposal of the International Society for Cutaneous Lymphomas (ISCL) and the cutaneous lymphoma task force of the European Organization of Research and Treatment of Cancer (EORTC). Blood.

[13] Marvi U, Chung L, Fiorentino DF (2012). Clinical presentation and evaluation of dermatomyositis. Indian J Dermatol.

[14] Ellgehausen P, Elsner P, Burg G (1998). Drug-induced lichen planus. Clin Dermatol.

[15] Iraji F, Faghihi G, Asilian A, Siadat AH, Larijani FT, Akbari M (2011). Comparison of the narrow band UVB versus systemic corticosteroids in the treatment of lichen planus: a randomized clinical trial. J Res Med Sci.

[16] Alsenaid A, Alamri A, Prinz JC, Ruzicka T, Wolf R (2016). Lichen planus of the lower limbs: successful treatment with psoralen cream plus ultraviolet A photochemotherapy. Dermatol Ther.

